# Escapism-Based Motivation Affected the Psychological Performances of High-Risk Internet Gaming Disorder Individuals

**DOI:** 10.3389/fpsyt.2022.855631

**Published:** 2022-03-14

**Authors:** Shuai Wang, Jialing Li, Siyu Wang, Can Mi, Wei Wang, Zhengjia Xu, Wenjing Xiong, Longxing Tang, Yanzhang Li

**Affiliations:** ^1^School of Psychology, Chengdu Medical College, Chengdu, China; ^2^School of Clinical Medicine, Chengdu Medical College, Chengdu, China

**Keywords:** escapism-based motivation, high risk of internet gaming disorder, psychological assessment, eye-tracking test, addiction – computational neuroscience

## Abstract

**Background:**

Escapism-based motivation (EBM) is considered as one of the diagnostic criteria for internet gaming disorder (IGD). However, how EBM affects the high risk of IGD (HIGD) population remains unclear.

**Methods:**

An initial number of 789 college students participated in the general, internet gaming behavior, and motivation surveys. After multiple evaluations, 57 individuals were identified as HIGD (25 with EBM, H-EBM; 32 with non-EBM, H-nEBM). In addition, 51 no-gaming individuals were included as the control group (CONTR). The cohorts completed the psychological assessments and eye-tracking tests, and analyses of group differences, correlations, and influencing factors of the indicators were performed.

**Results:**

The Barratt impulsiveness score of H-nEBM and H-EBM was significantly higher than that of CONTR (*MD* = 3.605, *P* = 0.017; *MD* = 3.744, *P* = 0.022). In addition, emotional intelligence self-emotion management ability was significantly lower in the H-EBM than in CONTR (*MD* = –2.038, *P* = 0.004). Correct rates and reaction times in the anti-saccade task differed significantly between the three groups (*F* = 3.525, *P* = 0.033; *F* = 4.459, *P* = 0.014). However, no differences were found in the comparison of the digital span test (DST), trail making test (TMT), animal verbal fluency test, Stroop test, and mental rotation test results. The anti-saccade test indicators were positively correlated with the DST results but negatively correlated with the Stroop test results (*P* < 0.05). Correct rates in the mental rotation test were negatively correlated with the TMT results but positively correlated with the DST results (*P* < 0.05). The participants with high Stroop test scores and no lover experience and who were raised by their grandparents were likely to develop EBM to engage in high risk of internet gaming disorder (*P* < 0.05).

**Conclusion:**

EBM has a significantly negative effect on impulsivity, self-emotion management ability, and response inhibition in the HIGD participants.

## Introduction

Currently, the number of internet gamers worldwide is estimated at one billion, with the farthest internet gaming reach observed in the population of China, South Korea, and Japan. Internet gaming audiences are projected to surpass 1.3 billion by 2025 (1). Internet gaming emerged as a popular but controversial online activity for individuals, from minors and seniors, owing to its potential effects on psychological health and wellbeing (2). The increasing popularity of internet gaming can be attributed mainly to severe social environments and the growing diversity of video games, which evolved rapidly from simple point-and-click to highly enticing roleplaying games in virtual worlds (3). Gamers can create representative characters or avatars in games to face situations they are unable to deal with in real life and further escape from their unsatisfactory lives, such as excessive pressure at school or work, strained interpersonal relationships, and so on (4). Whether internet games are beneficial to individual development is a controversial topic. Researchers believe that internet games can improve players’ spatial capabilities and responsiveness and reduce stress (5).

However, the most unfavorable effect of internet gaming is players’ unknowing progression toward a disease state known as internet gaming disorder (IGD). IGD was included in the third section of the latest (fifth) edition of the Diagnostic and Statistical Manual for Mental Disorders (DSM-5) and 11th revision of the International Classification of Diseases. This inclusion dramatically increased academic discussions and public concern about the importance of examining IGD. This gaming disorder can be observed in 3.05 and 5.0% of the population worldwide and in China, respectively (6). Individuals with IGD typically show symptoms related to a number of psychological and health problems, including depression, social anxiety, fatigue, loneliness, low self-esteem, and impulsivity. IGD patients exhibit high error rates in the case of game-related images in anti-saccade tasks, thereby revealing a dysfunctional attentional bias (7). Furthermore, IGD co-occurs with various psychiatric conditions and can lead to a range of negative outcomes.

Internet gaming disorder is a continuous development process, from not playing games to occasional to high-frequency playing and finally, out-of-control behavior (8). Therefore, the diagnosis for individuals who exhibit some symptoms but do not fully meet the IGD diagnostic criteria must not only judge whether the disorder reached the disease state but also establish awareness of early recognition and intervention (9). Such individuals are seen as high risk of IGD (HIGD). Although there is no unified definition of HIGD till now, some scales were successfully used in the screening of HIGD, such as the nine-item Internet Gaming Disorder Scale-Short Form (IGDS9-SF) (10), Internet Gaming Disorder Questionnaire (IGDQ) (11), and Chen Internet Addiction Scale (CIAS) (12). According to epidemiological surveys in various countries, the proportion of the HIGD population in the total population is approximately 9.0–11.5%, which is significantly higher than that of IGD sufferers (13, 14). Role-playing and other types of games can significantly increase the risk of HIGD (14). Ji et al. (15) observed the instantaneous frequency distribution of respiratory signals in 19 HIGD cases and believed that abdominal breathing training can help reduce this index, thereby reflecting the dynamic change process of sufferers’ psychophysiology. However, whether HIGD shares the aforementioned potential with IGD cognitive neural mechanisms and whether the degree of damage is reduced remain unclear.

The attraction of internet games appears to lie in their potential to satisfy different psychological needs, which can be conceptualized as motivation for gaming (16). Escapism-based motivation (EBM) is one of the most important online gaming motivations and belongs under the immersion component (17). Gamers with EBM use online environments to avoid real-life problems. Psychiatric symptoms in patients with IGD are significantly and strongly associated with EBM (16, 18). In addition, the Brief symptom inventory global severity index indicates that psychiatric distress levels have a significant positive direct effect and significant indirect (mediating) effect on problematic internet gaming and EBM (16). Hence, EBM is among the nine diagnostic criteria for IGD in the DSM-5. However, whether and how EBM affects HIGD individuals have yet to be fully understood.

Internet gaming is popular among college students in China, most of whom have not reached the disease state but can be considered as HIGD. Effective screening and detection as well as early intervention of HIGD, will help prevent them from developing into an addictive state. However, there is few studies to investigate the characteristics of HIGD, especially the role that motivation plays in the development of disease state. Hence, recognizing the psychological characteristics of HIGD students and the role of EBM is important step to detection HIGD individual. This study conducted a psychological assessment and eye-tracking analysis on HIGD students to verify the effects of EBM and examine the characteristics of this cohort.

## Materials and Methods

### Participants, General Information, and Internet Gaming Behavior Assessment

**FIGURE 1:**
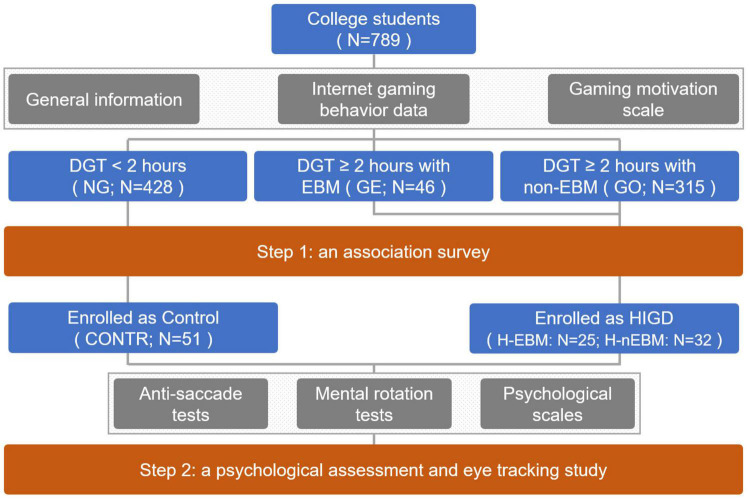
Participants’ flow.

A large-scale online survey was conduct to recruit internet gaming college students (between the ages of 18 and 25 years) and controls through an online questionnaire system (Wen Juan Xing^1^). Inclusion criteria were unlimited. However, the exclusion criteria were set as: (1) serious psychological problems, such as depression and anxiety; and (2) definitized mental disorder or its family history, such as schizophrenia, bipolar disorder, substance addiction, and so on. A total of 789 individuals met these criteria and answered all the survey questions completely, including those about general information and internet gaming behavior characteristics. The mean age of the sample was 20.78 ± 3.72 years, and 276 identified as male, whereas 513 identified as female. The participants’ flow is detailed in Figure 1.

The participants’ general information, including demographics, academic performance, upbringing, residence, and love experience, was collected with a self-administered questionnaire. Information on internet gaming behavior, including daily gaming time (DGT), gaming motivation, main gaming type, gaming history, and gaming equipment, was also collected. The individuals with an average DGT of ≥2 h in the past 3 months were classified as long-time players (19). Hence, in this study, the participants were divided into long-time players (DGT of ≥2 h) and short-time players (DGT of < 2 h, including the no-gaming individuals). Only the long-time players were asked to answer the remaining questions on internet gaming behavior. Motivation was evaluated using the escape subscale of the Online Game Motivation Scale (17), which had a Cronbach’s α of 0.85 in the Chinese college student population. The escape subscale included three items, with a total score of 15 points. The participants with a score over 8 were considered as gamers with EBM (20), and the participants with an IGDQ (11) score of ≥5 and a CIAS (12) score of ≥64 were considered as HIGD.

A total of 428 participants were identified as short-time players and set as the NG group. Among the 361 participants with a DGT of ≥2 h, the proportion with EBM to play internet games was 12.74% (*N* = 46; set as the GE group), and 315 participants demonstrated no significant EBM (set as the GO group). After IGDQ and CIAS evolutions, the 25 participants in the GE and 32 participants in the GO groups were considered as HIGD individuals and then set as HIGD-EBM group (H-EBM) and HIGD-non-EBM group (H-nEBM), respectively. In addition, 51 no-gaming individuals in this cohort voluntarily participated as the Control group (CONTR). Only individuals in H-EBM, H-nEBM, and CONTR groups needed to finish the following psychology assessments and eye-tracking trails.

### Psychological Assessments

The 108 participants were assessed using the trail making test (TMT), digital span test (DST), animal verbal fluency test (AFT), Stroop color and word test (Stroop), Barratt impulsiveness scale in the 11th (BIS) with 0.76 of Cronbach’s α and 0.85 of Kappa coefficient (21), and the Wong and Law Emotional Intelligence Scale (WLEIS) with 0.86 of Cronbach’s α and 0.78 of Kappa coefficient (22) to measure and screen their changed characteristics in recognition, affection, and behavior.

### Eye-Tracking Tests

The EyeLink 1000 system (SR Research Ltd., Canada) was used for the video-based eye-tracking tests. Dominant eye movements were recorded at a sampling rate of 1,000 Hz. With the Experiment Builder software (SR Research Ltd., Canada), the anti-saccade and mental rotation tasks were presented on a computer monitor (1,024 × 768 resolution, 60 Hz refresh rate) at a distance of 50 cm from the participants’ eyes.

#### Anti-saccade Task

**FIGURE 2:**
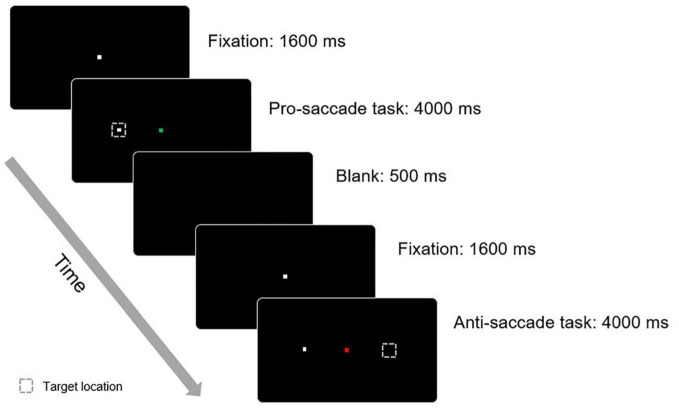
Design of anti-saccade task.

The task design included full pro-saccade and anti-saccade trials (Figure 2). At the start of each trial, the 108 participants were asked to fixate on a neutral stimulus (a white square with a visual angle of approximately 2°) that appeared at the center of the screen. The neutral fixation stimulus changed to an instructional cue indicating a pro-saccade or an anti-saccade when the participants maintained central fixation for 1,600 ms without generating a saccade. The instructional cues were colored pictograms of the same size as the neutral stimulus. A green square indicated that a pro-saccade should be made, whereas a red square indicated that an anti-saccade was required. At the same time, a peripheral target (a white square with a visual angle of approximately 2°) appeared at the left or right side of the screen at a distance of 5 or 10 cm from the instructional cue. The participants had 4,000 ms to execute the appropriate pro-saccade (look toward the target location) or anti-saccade (look away from the target in the equal but opposite direction) based on the instructional cue presented in the trial. When a trial was completed, a blank (without a fixation, cue, or target) appeared for 500 ms. A total of 64 trials (1:1 for pro- and anti-saccades, randomly presented) were designed for the execution components. The direction correct rate was set as the primary measure for this task performance, and the correct reaction time, fixation count, and saccade count were set as the secondary outcomes.

#### Mental Rotation Task

**FIGURE 3:**
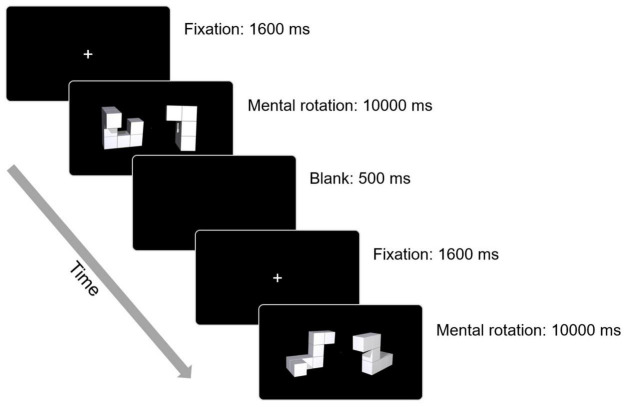
Design of mental rotation task.

The mental rotation task consisted of 20 unique block pairs resulting in a 50% “same” and 50% “different” result. The stimuli were presented pairwise in two different angular disparities of 45° and 90°, and each figure had a dimension of 400 × 400 pixels. A fixation stimulus (a white cross) appeared at the center of the screen at the start of each trial. The neutral fixation stimulus changed to a task trial when the participants maintained central fixation for 1,600 ms. Within 10,000 ms in each trial, the participants must determine the final judgment and press “Y” for “same” and “N” for “different.” When a trial was completed, a blank appeared for 500 ms. The task design is presented in Figure 3. The reaction correct rate was set as the primary measure for this task performance, and the correct reaction time, fixation count, and saccade count were set as the secondary outcomes.

### Data and Statistical Analyses

The eye-tracking test data were extracted using DataViewer (SR Research Ltd., Canada). The general information and internet gaming behavior assessment and psychological assessment data were manually extracted from the original questionnaires. All the statistical analyses were performed using SPSS 22.0 (IBM Co., United States). To examine the differences in the DGT and motivation catastrophizing, demographic and internet gaming behavioral characteristic, and questionnaire data between the groups, *X*^2^ and *t*-tests were used for the categorical and continuous variables, respectively. To examine the differences in the anti-saccade correct rates and reaction times, a series of repeated analysis of variance (ANOVA) measures was performed, with trial type (anti-saccade vs. pro-saccade), direction (left vs. right), and amplitude (5 cm vs. 10 cm) as the within-group factors and group (H-EBM vs. H-nEBM vs. CONTR) as the between-group variable. To quantify the effect size of the observed results, the partial eta-squared η_*p*_^2^ was calculated. Logistic regression was performed to further obtain the associated factors in the HIGD participants with EBM, and *P* < 0.05 results were considered as statistically significant.

## Results

### Differences in General Characteristics

As shown in Table 1, gender, upbringing, and residence distributions differed significantly between the GE, GO, and NG groups (*X*^2^ = 68.921, *P* < 0.001; *X*^2^ = 12.222, *P* = 0.016; *X*^2^ = 8.052, *P* = 0.018). The proportion of the male participants was significantly higher in the GE group than in the NG group (*X*^2^ = 28.950, *P* < 0.001). The proportion of the participants raised by their grandparents was significantly higher in the GE group than in the GO group (*X*^2^ = 8.811, *P* = 0.012). Furthermore, the proportion of urban residents was significantly higher in the GO group than in the NG group (*X*^2^ = 8.020, *P* = 0.005). No significant differences were observed in age, main gaming type, gaming history, and gaming equipment between the three groups or between the GE and GO groups (all *P* > 0.05).

**TABLE 1:** Association of the general characteristics with gaming experiences and motivations.

Characteristics	GE (*N* = 46)	GO (*N* = 315)	NG (*N* = 428)	*F*/*X*^2^	*P*
Age	20.55 ± 4.29	20.81 ± 3.51	20.77 ± 3.68	0.319	0.652
Gender (Male/Female, *N*)	27/19	154/161	95/333^#^	68.921	**<0.001**
Academic performance (Excellent/Fair/Poor, *N*)	6/32/8	40/247/28	57/342/29^#^	6.575	0.160
Upbringing (Parents/Grandparents/Other, *N*)	39/6/1	301/11/3^#^	386/29/13	12.222	**0.016**
Residence (Urban/Rural, *N*)	21/25	167/148	182/246^※^	8.052	**0.018**
Love experience (Yes/No, *N*)	24/22	174/141	204/224	4.195	0.123
Main gaming type (Role play/Other, *N*)*	24/22	160/155	–	0.031	0.861
Gaming history (<2y/ ≥2y, *N*)*	16/30	115/200	–	0.052	0.820
Gaming equipment (Phone/Computer/Pad, *N*)*	34/11/1	241/63/11	–	0.545	0.761

*Participants of daily gaming time ≥ 2 h. ^#^Compared with GE, the difference was statistically significant. ^※^Compared with GO, the difference was statistically significant.

*Bold values mean P < 0.05.*

### Differences in Psychological Results

**FIGURE 4:**
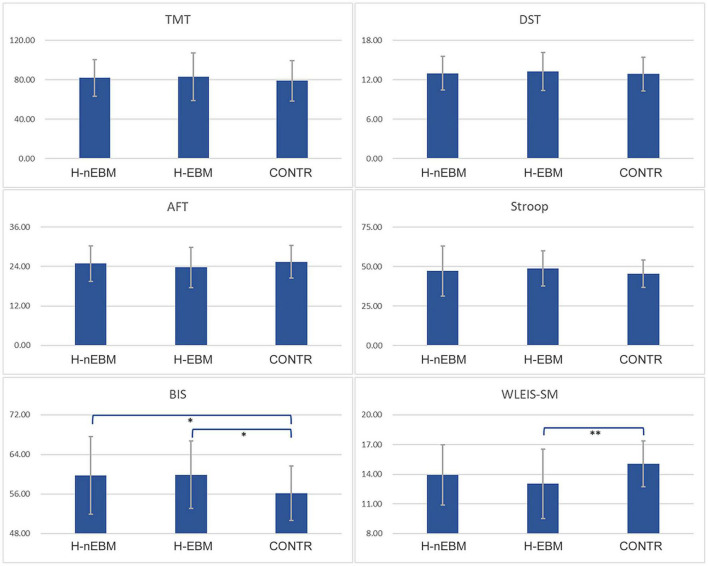
Psychological assessment results of 108 participants. ^∗^*P* < 0.05 and ^∗∗^*P* < 0.001.

A total of 57 HIGD and 51 control respondents (H-EBM = 25, H-nEBM = 32, CONTR = 51) participated in the psychological assessments. No significant differences were found between the three groups in the comparison of the TMT, DST, AFT, and Stroop test results (all *P* > 0.05; Figure 4). The BIS index was significantly higher in the H-nEBM and H-EBM than in the CONTR (*MD* = 3.605, *P* = 0.017; *MD* = 3.744, *P* = 0.022; Figure 4). Furthermore, self-emotion management in the WLEIS was significantly lower in the H-EBM individuals than in the CONTR (*MD* = –2.038, *P* = 0.004; Figure 4).

### Differences in Eye-Tracking Results

A total of 57 HIGD and 51 control respondents (H-EBM = 25, H-nEBM = 32, CONTR = 51) participated in the eye-tracking tests. As shown in Table 2, the correct rates and reaction times in the anti-saccade task differed significantly between the three groups (*F* = 3.525, *P* = 0.033; *F* = 4.459, *P* = 0.014). The H-nEBM had significantly lower correct rates (*MD* = –0.126, *P* = 0.010), longer reaction times (*MD* = 176.398, *P* = 0.007), and more fixation (*MD* = 1.776, *P* = 0.033) and saccade counts (*MD* = 1.825, *P* = 0.028) than the CONTR. Furthermore, the H-EBM had significantly shorter reaction times than the H-nEBM (*MD* = –187.01, *P* = 0.016). However, no significant differences were observed in the correct rates, reaction times, and fixation and saccade count in the mental rotation task between the three groups (all *P* > 0.05).

**TABLE 2:** The results of eye-tracking tests.

Indicators	H-EBM (*N* = 25)	H-nEBM (*N* = 32)	CONTR (*N* = 51)	*F*	*P*
Anti-saccade task					
Correct rate (%)	0.37 ± 0.22	0.31 ± 0.17	0.43 ± 0.23246^※^	3.525	**0.033**
Reaction time (ms)	2044.27 ± 271.32	2231.28 ± 315.14^#^	2054.88 ± 274.12246^※^	4.459	**0.014**
Fixation count (times)	4.52 ± 2.08	5.62 ± 6.38	3.84 ± 0.67246^※^	2.347	0.101
Saccade count (times)	3.59 ± 2.11	4.73 ± 6.37	2.90 ± 0.70246^※^	2.480	0.089
**Mental rotation task**					
Correct rate (%)	0.80 ± 0.19	0.81 ± 0.16	0.76 ± 0.17	0.910	0.406
Reaction time (ms)	4431.76 ± 121.20	5110.43 ± 159.60	4804.66 ± 139.07	1.609	0.205
Fixation count (times)	17.07 ± 5.00	18.94 ± 5.36	18.02 ± 4.36	1.054	0.352
Saccade count (times)	16.35 ± 5.02	18.24 ± 5.40	17.27 ± 4.37	1.080	0.343

*^#^Compared with H-EBM, the difference was statistically significant.*

*^※^Compared with DGT ≥ 2 h with H-nEBM, the difference was statistically significant. Bold values mean P < 0.05.*

**FIGURE 5:**
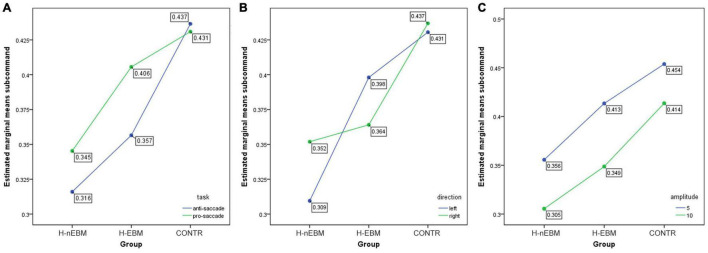
Differences in correct rates in anti-saccade task between H-EBM, H-nEBM, and CONTR groups in **(A)** pro-saccade and anti-saccade trials; **(B)** left and right directions; **(C)** 5 and 10 cm amplitudes.

**FIGURE 6:**
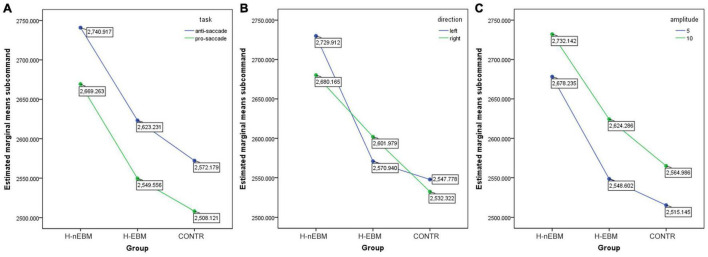
Differences in reaction times in anti-saccade task between H-EBM, H-nEBM, and CONTR groups in **(A)** pro-saccade and anti-saccade trials; **(B)** left and right directions; **(C)** 5 cm and 10 cm amplitudes.

For the anti-saccade task, a 3 (group: H-EBM vs. H-nEBM vs. CONTR) × 2 (trial: pro-saccade vs. anti-saccade or direction: left vs. right or amplitude: 5 cm vs. 10 cm) ANOVA was performed. As shown in Figure 5, an amplitude of 5 cm, compared with an amplitude of 10 cm, was associated with higher correct rates [*F*(1,108) = 15.862, *P* < 0.001, η_*p*_^2^ = 0.003], thereby revealing a significant main effect for amplitude. The trial type and direction were not significant for this parameter [trial: *F*(1,108) = 3.485, *P* = 0.062, η_*p*_^2^ = 0.001; direction: *F*(1,108) = 0.143, *P* = 0.705, η_*p*_^2^ < 0.001]. No interaction was found in the correct rates (all *P* > 0.05). As depicted in Figure 6, the anti-saccade trial (compared with the prosaccade trial) and an amplitude of 10 cm (compared with an amplitude of 5 cm) were associated with longer reaction times [trial: *F*(1,108) = 19.139, *P* < 0.001, η_*p*_^2^ = 0.003; amplitude: *F*(1,108) = 14.042, *P* < 0.001, η_*p*_^2^ = 0.002], thereby revealing a significant main effect for trial type and amplitude. Direction was insignificant for this parameter [*F*(1,108) = 0.508, *P* = 0.476, η_*p*_^2^ < 0.001]. No interaction was found in the reaction times (all *P* > 0.05).

### Correlation Analysis in Escapism-Based Motivation

Fixation and saccade count in the anti-saccade test were positively correlated with the DST results (*r* = 0.543, *P* = 0.005; *r* = 0.541, *P* = 0.005). Reaction times, fixation counts, and saccade counts in the anti-saccade test were negatively correlated with the Stroop test results (*r* = –0.420, *P* = 0.037; *r* = –0.475, *P* = 0.016; *r* = –0.474, *P* = 0.017). Correct rates in the mental rotation test were negatively correlated with the TMT results (*r* = –0.514, *P* = 0.009) but positively correlated with the DST results (*r* = 0.403, *P* = 0.046).

### Logistic Regression Analysis

The participants with high Stroop test scores were 1.088 times more likely to be HIGD with EBM than their counterparts (*Wald* = 4.776, *P* = 0.029, 95% CI: 1.009–1.173). The participants with satisfactory academic performance were 0.064 times more likely to engage in gaming behavior than those with poor academic performance (*Wald* = 5.517, *P* = 0.019, 95% CI: 0.006–0.635). The participants raised by their parents had a significantly lower probability of engaging in gaming behavior than those raised by other family members (*Wald* = 132.042, *P* < 0.001, 95% CI: 0.000–0.000). Furthermore, the participants with no lover experience were 11.157 times more likely to engage in gaming behavior than those with lover experience (*Wald* = 7.282, *P* = 0.007, 95% CI: 1.935–64.330). The detailed results are presented in Table 3.

**TABLE 3:** Logistic regression of occurring HIGD with EBM.

Variables	*SE*	*Wald*	*P*	*OR* (*95%CI*)
Age	0.305	1.908	0.167	0.657 (0.361∼1.193)
TMT	0.019	1.814	0.178	1.026 (0.988∼1.066)
DST	0.138	2.273	0.132	1.231 (0.940∼1.612)
AFP	0.074	0.923	0.337	0.931 (0.805∼1.077)
**Stroop**	**0.038**	**4.776**	**0.029**	**1.088 (1.009∼1.173)**
BIS	0.066	2.418	0.120	1.107 (0.974∼1.259)
WLEIS	0.062	2.558	0.110	1.105 (0.978∼1.248)
**Gender**				
Female	Ref.			
Male	0.758	0.287	0.592	0.666 (0.151∼2.941)
**Academic performance**				
Poor	Ref.			
Excellent	9214.551	0.000	0.998	0.000 (0.000∼0.000)
**Fair**	**1.170**	**5.517**	**0.019**	**0.064 (0.006∼0.635)**
**Upbringing**				
Other	Ref.			
**Parents**	**2.141**	**132.042**	**<0.001**	**0.000 (0.000∼0.000)**
**Residence**				
Rural	Ref.			
Urban	0.777	0.018	0.892	1.111 (0.243∼5.091)
**Love experience**				
Yes	Ref.			
**No**	**0.894**	**7.282**	**0.007**	**11.157 (1.935∼64.330)**

*Bold values mean P < 0.05.*

## Discussion

In this study, we observed the pleiotropic effects of EBM on HIGD college students by conducting psychological assessments and eye-tracking tests. The participants with long-time internet gaming experience and EBM were mostly male, raised by their grandparents, and demonstrated higher impulsivity and lower self-emotion management ability than the healthy controls or the participants with long-time internet gaming experience with non-EBM. In an online survey of the Korean population, the individuals with IGD were mostly male and exhibited dysfunctional impulsivity (23). The male gender and impulsiveness are considered as risk factors for IGD (24, 25). EBM is a predictor of IGD (26). However, in long-time but not addiction internet gamers, the factors inducing the association between EBM and the male gender and impulsiveness and the role of long-time internet gaming behavior in the relationship between the two factors remain unknown. Grandparents raising their grandchildren is common in China; thus, the monitoring of children’s behavioral and emotional difficulties is generally lacking (27, 28). When children become accustomed to playing online games without restriction, they may suffer from online gaming addiction, which is indicated by their tendency to continue playing and ignore social obligations and reality (29). Thus, the result of the considerable proportion of the long-time internet gamers, especially those with EBM, being raised by their grandparents can be explained easily. In addition, the logistic regression analysis revealed that being raised by parents and having satisfactory academic performance were low-risk factors, whereas lack of lover experience was a high-risk factor for HIGD and EBM.

The anti-saccade task is among the methods used to measure response inhibition, which is an important executive control function component (30). Patients with attention-deficit/hyperactivity disorder (31), schizophrenia (32), or IGD (33) show significant deficits in response inhibition and abnormal performance in anti-saccade and/or Stroop tasks. In a previous eye-tracking study with 23 IGD patients and 27 healthy controls, in the anti-saccade task, the IGD group exhibited higher error rates in the case of game-related images compared with neutral or scrambled images (7). An independent component analysis of a probability discounting task showed that IGD patients preferred risky over fixed options and demonstrated shorter reaction times in their behavioral results and less engagement in the executive control network compared with healthy controls, thereby suggesting deficits in the executive control function of IGD patients (34). In the present study, the HIGD participants with EBM, and especially those with non-EBM, had low correct rates in the anti-saccade task. Furthermore, the latter participants had longer reaction times than the healthy controls. However, both groups exhibited improved performance in the short-amplitude and prosaccade trials. These results suggested that non-EBM may induce a response inhibition deficit in the HIGD participants. Therefore, the executive control function of the HIGD individuals may have been impaired before they developed an internet gaming addiction. However, whether different degrees of executive control function impairment exist between IGD and HIGD individuals has yet to be reported.

In the correlation analysis, the anti-saccade results were mostly negatively correlated with the Stroop test results and positively correlated with the DST results. As stated previously, the Stroop effect describes the delay in the reaction time between congruent and incongruent stimuli. The Stroop test is widely used in clinical practice and investigations to measure individuals’ selective attention capacity and skills as well as their executive control function (35). In Stroop test results, the higher the score, the stronger the executive control function. However, in the anti-saccade task, the shorter the reaction time, and the less the fixation count or saccade count, the stronger the executive control function. The DST is a simple behavioral measure of working memory capacity, which is the cognitive ability to store and manage information on a transient basis (36). Working memory capacity and executive functions share a common underlying executive attention component that is strongly predictive of high-level cognition (37). Hence, working memory capacity may be associated with executive functions. The positive correlation between the two factors measured by the anti-saccade task and DST in the present study can be explained easily.

Mental rotation is defined as the ability to rotate mental representations of two-dimensional and three-dimensional objects and related to the visual representation of such rotations in the human mind (38). A relationship exists between spatial processing cognitive rates, general intelligence, and mental rotation (39). A previous experiment showed that children’s initial low mental rotation performance improves after playing computer games requiring mental rotation skills (40). A subsequent investigation also demonstrated that playing computer games improves mental rotation scores in general, and women’s gains are significantly greater than those of men, and the most significant gains are accomplished when practice is accumulated (41). These findings implied that computer-based games could be used in schools to enhance children’s spatial abilities. However, in the present study, the mental rotation performance of the three groups demonstrated no significant differences, thereby suggesting that EBM had no effect on the spatial abilities of the HIGD participants. The inconsistent conclusions above may be attributed to different research populations and game types.

Several limitations of this study should be addressed. The sample for step 2 was selected from the sample in step 1 based on the respondents’ willingness to participate in the subsequent tests; thus, selection bias may exist. In addition, other motivations collectively referred to as non-EBM in this study may be potential confounding factors affecting the accuracy of the results. Despite such limitations, EBM showed a significantly negative effect on impulsivity, self-emotion management ability, and response inhibition in the HIGD college students. Finally, lover experience and parental upbringing may help college students avoid developing EBM.

## Conclusion

Escapism-based motivation has a significantly negative effect on impulsivity, self-emotion management ability, and response inhibition in the HIGD participants. These results will help us to understand the psychological characteristics of HIGD and early identify this population, which would help for prevention of IGD.

## Data Availability Statement

The original contributions presented in the study are included in the article/supplementary material, further inquiries can be directed to the corresponding author.

## Ethics Statement

The studies involving human participants were reviewed and approved by the Medical Ethics Committee of Chengdu Medical College. The patients/participants provided their written informed consent to participate in this study.

## Author Contributions

All authors contributed to the data analysis, drafting, revision of this manuscript, agreed on the journal to which the article will be submitted, gave final approval of the version to be published, and agreed to be accountable for all aspects of the work.

## Conflict of Interest

The authors declare that the research was conducted in the absence of any commercial or financial relationships that could be construed as a potential conflict of interest.

## Publisher’s Note

All claims expressed in this article are solely those of the authors and do not necessarily represent those of their affiliated organizations, or those of the publisher, the editors and the reviewers. Any product that may be evaluated in this article, or claim that may be made by its manufacturer, is not guaranteed or endorsed by the publisher.

## Footnotes

1
www.wjx.cn

## References

[1] Clement J. *Online Gaming in the U.S. and Worldwide.* New York, NY: Statista (2020).

[2] Slater A Halliwell E Jarman H Gaskin E. More than just child’s play?: an experimental investigation of the impact of an appearance-focused internet game on body image and career aspirations of young girls. *J Youth Adolesc.* (2017) 46:2047–59. 10.1007/s10964-017-0659-7 28316057 PMC5561163

[3] McAllister G White GR. Video game development and user experience. In: Bernhaupt R editor. *Game User Experience Evaluation.* Cham: Springer International Publishing (2015). p. 11–35.

[4] Allen JJ Anderson CA. Satisfaction and frustration of basic psychological needs in the real world and in video games predict internet gaming disorder scores and well-being. *Comput Hum Behav.* (2018) 84:220–9. 10.1016/j.chb.2018.02.034

[5] Subrahmanyam K Greenfield P Kraut R Gross E. The impact of computer use on children’s and adolescents’ development. *J Appl Dev Psychol.* (2001) 22:7–30.

[6] Stevens MW Dorstyn D Delfabbro PH King DL. Global prevalence of gaming disorder: a systematic review and meta-analysis. *Aust N Z J Psychiatry.* (2021) 55:553–68. 10.1177/0004867420962851 33028074

[7] Kim M Lee TH Choi JS Kwak YB Hwang WJ Kim T Dysfunctional attentional bias and inhibitory control during anti-saccade task in patients with internet gaming disorder: an eye tracking study. *Prog Neuropsychopharmacol Biol Psychiatry.* (2019) 95:109717. 10.1016/j.pnpbp.2019.109717 31351161

[8] Lu L Yu X Li JT Wang GH Wang G Li T *Standards for Diagnosis and Treatment of Mental Disorders (2020 Edition).* Beijing: National Health Commission (2020).

[9] Xiang YT Jin Y Zhang L Li L Ungvari GS Ng CH An overview of the expert consensus on the prevention and treatment of gaming disorder in China (2019 edition). *Neurosci Bull.* (2020) 36:825–8. 10.1007/s12264-020-00475-w 32125603 PMC7340689

[10] Macur M Pontes HM. Internet gaming disorder in adolescence: investigating profiles and associated risk factors. *BMC Public Health.* (2021) 21:1547. 10.1186/s12889-021-11394-4 34384394 PMC8361866

[11] Jeromin F Rief W Barke A. Validation of the internet gaming disorder questionnaire in a sample of adult German-speaking internet gamers. *Cyberpsychol Behav Soc Netw.* (2016) 19:453–9. 10.1089/cyber.2016.0168 27428033

[12] Mak KK Lai CM Ko CH Chou C Kim DI Watanabe H Psychometric properties of the revised chen internet addiction scale (CIAS-R) in Chinese adolescents. *J Abnorm Child Psychol.* (2014) 42:1237–45. 10.1007/s10802-014-9851-3 24585392

[13] Gentile DA Choo H Liau A Sim T Li DD Fung D Pathological video game use among youths: a two-year longitudinal study. *Pediatrics.* (2011) 127:E319–29. 10.1542/peds.2010-1353 21242221

[14] Han H Jeong H Jo SJ Son HJ Yim HW. Relationship between the experience of online game genre and high risk of internet gaming disorder in Korean adolescents. *Epidemiol Health.* (2020) 42:e2020016. 10.4178/epih.e2020016 32272007 PMC7285446

[15] Ji H-M Hsiao T-C. Instantaneous respiratory response of gamer with high-risk internet gaming disorder during game-film stimuli by using complementary ensemble empirical mode decomposition. In: *Proceedings of the 2020 42nd Annual International Conference of the IEEE Engineering in Medicine & Biology Society (EMBC).* Montreal, QC (2020). p. 980–3. 10.1109/EMBC44109.2020.9175719 33018149

[16] Király O Urbán R Griffiths MD Ágoston C Nagygyörgy K Kökönyei G The mediating effect of gaming motivation between psychiatric symptoms and problematic online gaming: an online survey. *J Med Int Res.* (2015) 17:e88. 10.2196/jmir.3515 25855558 PMC4405620

[17] Yee N. Motivations for play in online games. *Cyberpsychol Behav.* (2006) 9:772–5. 10.1089/cpb.2006.9.772 17201605

[18] Zhong Z-J Yao MZ. Gaming motivations, avatar-self identification and symptoms of online game addiction. *Asian J Commun.* (2013) 23:555–73. 10.1080/01292986.2012.748814

[19] Jaruratanasirikul S Wongwaitaweewong K Sangsupawanich P. Electronic game play and school performance of adolescents in southern Thailand. *Cyberpsychol Behav.* (2009) 12:509–12. 10.1089/cpb.2009.0035 19594380

[20] Dong R Fu Y Hou X Yu L. The mediating role of escape motivation and flow experience between frustration and online game addiction among university students. *Chin J Behav Med Brain Sci.* (2021) 30:327–32.

[21] Yang H-Q Yao S-Q Zhu X-Z Auerbach RP Abela JR Tong X. The Chinese version of the Barratt impulsiveness scale, 11th version (BIS-11) in adolescents: its reliability and validity. *Chin J Clin Psychol.* (2007) 15:4–6. 10.1046/j.1440-1819.2001.00796.x 11285088

[22] Shi J Wang L. Validation of emotional intelligence scale in Chinese university students. *Pers Individ Differ.* (2007) 43:377–87.

[23] Rho MJ Lee H Lee TH Cho H Jung DJ Kim DJ Risk factors for internet gaming disorder: psychological factors and internet gaming characteristics. *Int J Environ Res Public Health.* (2017) 15:40. 10.3390/ijerph15010040 29280953 PMC5800139

[24] Gentile DA Bailey K Bavelier D Brockmyer JF Cash H Coyne SM Internet gaming disorder in children and adolescents. *Pediatrics.* (2017) 140(Suppl. 2):S81–5. 10.1542/peds.2016-1758H 29093038

[25] Severo RB Soares JM Affonso JP Giusti DA de Souza Junior AA de Figueiredo VL Prevalence and risk factors for internet gaming disorder. *Braz J Psychiatry.* (2020) 42:532–5. 10.1590/1516-4446-2019-0760 32785455 PMC7524423

[26] Ramos-Diaz J Ramos-Sandoval R Király O Demetrovics Z Griffiths MD. An exploratory study on motivational predictors in internet gaming disorder among Peruvian gamers. In: *Proceedings of the 2018 IEEE Sciences and Humanities International Research Conference (SHIRCON).* Lima (2018). p. 1–4.

[27] Smith GC Palmieri PA. Risk of psychological difficulties among children raised by custodial grandparents. *Psychiatr Serv.* (2007) 58:1303–10. 10.1176/ps.2007.58.10.1303 17914007 PMC2083282

[28] Chen F Liu G. The health implications of grandparents caring for grandchildren in China. *J Gerontol B Psychol Sci Soc Sci.* (2012) 67:99–112. 10.1093/geronb/gbr132 22156630 PMC3267025

[29] Anandari DR. Permissive parenting style and its risks to trigger online game addiction among children. In: *Proceedings of the Asian Conference 2nd Psychology & Humanity.* Nagoya: The International Academic Forum (2016). p. 773–81.

[30] Munoz DP Everling S. Look away: the anti-saccade task and the voluntary control of eye movement. *Nat Rev Neurosci.* (2004) 5:218–28. 10.1038/nrn1345 14976521

[31] Fernandez-Ruiz J Hakvoort Schwerdtfeger RM Alahyane N Brien DC Coe BC Munoz DP. Dorsolateral prefrontal cortex hyperactivity during inhibitory control in children with ADHD in the antisaccade task. *Brain Imaging Behav.* (2020) 14:2450–63. 10.1007/s11682-019-00196-3 31493141

[32] Enticott PG Ogloff JR Bradshaw JL. Response inhibition and impulsivity in schizophrenia. *Psychiatry Res.* (2008) 157:251–4. 10.1016/j.psychres.2007.04.007 17916385

[33] Argyriou E Davison CB Lee TTC. Response inhibition and internet gaming disorder: a meta-analysis. *Addict Behav.* (2017) 71:54–60. 10.1016/j.addbeh.2017.02.026 28264786

[34] Wang L Wu L Lin X Zhang Y Zhou H Du X Dysfunctional default mode network and executive control network in people with internet gaming disorder: independent component analysis under a probability discounting task. *Eur Psychiatry.* (2016) 34:36–42. 10.1016/j.eurpsy.2016.01.2424 26928344

[35] Lamers MJM Roelofs A Rabeling-Keus IM. Selective attention and response set in the stroop task. *Mem Cogn.* (2010) 38:893–904. 10.3758/Mc.38.7.893 20921102

[36] Conklin HM Curtis CE Katsanis J Iacono WG. Verbal working memory impairment in schizophrenia patients and their first-degree relatives: evidence from the digit span task. *Am J Psychiatry.* (2000) 157:275–7. 10.1176/appi.ajp.157.2.275 10671401

[37] McCabe DP Roediger HL McDaniel MA Balota DA Hambrick DZ. The relationship between working memory capacity and executive functioning: evidence for a common executive attention construct. *Neuropsychology.* (2010) 24:222–43. 10.1037/a0017619 20230116 PMC2852635

[38] Kawamichi H Kikuchi Y Ueno S. Spatio-temporal brain activity related to rotation method during a mental rotation task of three-dimensional objects: an MEG study. *Neuroimage.* (2007) 37:956–65. 10.1016/j.neuroimage.2007.06.001 17613250

[39] Hugdahl K Thomsen T Ersland L. Sex differences in visuo-spatial processing: an fMRI study of mental rotation. *Neuropsychologia.* (2006) 44:1575–83. 10.1016/j.neuropsychologia.2006.01.026 16678867

[40] De Lisi R Wolford JL. Improving children’s mental rotation accuracy with computer game playing. *J Genet Psychol.* (2002) 163:272–82. 10.1080/00221320209598683 12230149

[41] Cherney ID. Mom, let me play more computer games: they improve my mental rotation skills. *Sex Roles.* (2008) 59:776–86. 10.1007/s11199-008-9498-z

